# Robust multi-target multi-scale tomato leaf disease detection for precision agriculture applications

**DOI:** 10.3389/fpls.2026.1829521

**Published:** 2026-06-03

**Authors:** Jun-Zhang Pan, Yang Xie, Shuai-Yang Zhao, Yao-Lan Kang, Shu-Hui Yin, Zhen-Qi Fan, Li-Feng Guo, Li-Mei Qi, Chun-Jing Si

**Affiliations:** 1 College of Information Engineering, Tarim University, Alaer, China; 2 Hangzhou Zhenshan Integrity Information Technology Co., Ltd., Hangzhou, China; 3 School of Network and Information Security, Tarim University, Alaer, China; 4 Key Laboratory of Tarim Oasis Agriculture, Ministry of Education, Tarim University, Alaer, China

**Keywords:** CBAM attention, multi-target and multi-scale, tomato leaf disease detection, transfer learning, YOLO v8s

## Abstract

The tomato is one of the most important economic crops worldwide; frequent occurrences of foliar diseases can severely affect its quality and yield, resulting in substantial economic losses. However, state-of-the-art methods still struggle with multi-target, multi-scale disease detection in complex scenarios, lacking accuracy and speed for tomato leaf diagnosis. A novel improved YOLO v8s model is proposed in this study to achieve high-precision and fast identification of multi-target and multi-scale tomato leaf diseases. First, a multi-target, multi-scale image dataset encompassing seven typical tomato diseases was developed to effectively enhance the model’s robustness under complex practical scenario by integrating multiple public datasets and employing diverse data augmentation techniques. Second, a transfer learning strategy was employed to transfer high-quality features from a pretrained model to the disease detection task, thereby improving convergence speed and generalization ability. Finally, the CBAM (Convolutional Block Attention Module) channel-spatial attention mechanism was introduced into the YOLO v8s network, enabling the model to adaptively focus on critical regions and significantly enhance feature extraction and target localization performance. Experimental results demonstrate that the improved YOLOv8s-CBAM model achieves superior performance in complex scenarios, with a precision of 96.9%, recall of 97.3%, F1 score of 97.0%, and mAP@0.5 of 99.1%, representing improvements of 2.5%, 2.0%, 2.2%, and 1.8%, respectively, over the original YOLO v8s model. Moreover, the model size was reduced to 24.8 MB, a decrease of 11.7 MB compared to the original, achieving an effective balance between accuracy and lightweight design. These results indicate that the proposed method exhibits enhanced feature extraction and localization stability in multi-target, multi-scale disease identification tasks, providing an effective technical solution for automated detection in complex agricultural disease scenarios.

## 1 Introduction

Tomato is among the most important vegetable crops worldwide, serving as a major nutritional component of daily diets and a cornerstone raw material for the food-processing industry and international agricultural trade. As living standards rise and demand for high-quality produce increases, tomato cultivation has expanded and the sector has moved toward large-scale, modernized production. This expansion is accompanied by intense pest and disease pressure, with foliar diseases constituting a principal constraint on yield and quality. Bacterial spot, early blight, and late blight are widely distributed, highly transmissible, and strongly destructive, frequently causing substantial yield losses or even crop failure and thereby imposing significant economic burdens on farmers and supply chains (Savary et al., 2019). Critically, disease outbreaks often coincide with peak field operations, creating a pressing need for detection systems capable of rapid and accurate diagnosis under limited time and labor. Establishing robust, field-oriented disease detection is therefore essential not only to safeguard yield and quality but also to optimize pesticide use, reduce production costs and environmental load, and support the stable, high-value development of the tomato industry.

Deep learning techniques have been extensively studied and applied in the field of plant leaf disease identification (Saleem et al., 2019; Li et al., 2021). By integrating advanced algorithms of machine learning and computer vision, researchers can construct intelligent disease detection systems to achieve automated identification and accurate diagnosis of crop leaf diseases. This not only improves the efficiency of agricultural production but also provides technical support for precision agriculture, significantly reducing the cost and errors associated with manual detection. Furthermore, in the research on plant leaf disease classification, scholars continue to explore novel methods to enhance the accuracy and robustness of models in disease recognition.

The recognition and localization capabilities of relevant algorithms for leaf diseases have been continuously enhanced as object detection technology continues to advance. Early evidence from Mohanty et al., who achieved 99.35% accuracy on the PlantVillage repository (54, 306 images across 14 crops and 26 diseases), demonstrated the immense potential of end-to-end learning using deep CNNs for leaf imagery (Mohanty et al., 2016). This feasibility was further reinforced by Ferentinos, whose systematic comparison cemented CNNs as the de-facto baseline for agricultural vision (Ferentinos, 2018). Building on this successful foundation, modern one-stage YOLO detectors have emerged as the practical evolution for real-time field applications (Lawal, 2021). These detectors integrate multi-scale feature fusion and small-object-oriented data augmentation, most notably the mosaic augmentation popularized by YOLO v4, which synthesizes dense, cross-scale scenes similar to challenging field imagery (Kornblith et al., 2019). To further refine the features extracted by deep networks, lightweight attention modules have been developed to enhance lesion saliency. Techniques range from SENet for channel recalibration (Chen et al., 2020) and CBAM for sequential channel-then-spatial reweighting (Bochkovskiy et al., 2020a), to Coordinate Attention which injects position-aware channel attention (Hu et al., 2018). Notably, achieving precise detection of multiple plant leaf diseases in complex natural environments remains a challenging task (Liu and Wang, 2020).

In complex agricultural settings, particularly for tomato-specific scenes (MTMS), recent studies have successfully combined attention with multi-scale fusion to improve detection robustness (Woo et al., 2018). The reported accuracy gains from attention-enhanced detectors, such as SE-YOLO v5 for tomato virus detection, confirm the value of integrating attention into a YOLO-style framework (Hou et al., 2021; Zhang et al., 2021; Zhu et al., 2023). Consequently, these results motivate our selection of CBAM for attention-guided feature enhancement within our YOLO-based detector. Finally, transfer learning is crucial for accelerating model convergence and improving generalization, particularly in data-limited agricultural contexts. Kornblith et al. quantified that stronger ImageNet models tend to transfer better across downstream datasets, unequivocally supporting the use of pretrained backbones (Qi et al., 2022; Wang et al., 2024a). In alignment with this evidence, we selected the YOLO v8s family for its practical foundation, refined CSPDarknet-style backbone, C2f module, and anchor-free decoupled head (Ultralytics Docs, 2023). Our final methodology is thus an optimized synthesis: adopting YOLO v8s as the lightweight foundation, initializing it with pretrained weights to leverage transfer learning, and adding the CBAM module for attention-guided feature enhancement.

Although deep learning methods have achieved high accuracy, their application to object detection in agriculture faces significant limitations, particularly when addressing MTMS disease detection in complex field settings. These challenges stem from the nature of existing public datasets, which suffer from class imbalance and a scarcity of early-stage, small, or weak-texture lesions, biasing model optimization toward larger, salient targets. Such datasets often fail to represent the dense, cross-scale layouts and the co-occurrence of multiple targets in complex backgrounds found in real-world scenarios (Bochkovskiy et al., 2020b). Methodologically, even a highly efficient base architecture like YOLO v8s requires enhancement, as its standard feature extraction process struggles to effectively localize small or low-contrast targets which represents a critical flaw when addressing the scarcity of such lesions in existing datasets (Woo et al., 2018; Hu et al., 2020). Without dedicated mechanisms, models lack the ability to provide local channel and spatial reweighting to enhance lesion saliency and localization stability, a recognized challenge in complex settings like tomato disease detection (Qi et al., 2022; Wang et al., 2024b). Furthermore, training on limited agricultural imagery presents a fundamental difficulty, as models are susceptible to overfitting and poor generalization due to data scarcity, posing a significant risk to model robustness (Kornblith et al., 2019).

Specifically, practical deployments in tomato cultivation still confront severe bottlenecks regarding multi-objective and multi-scale detection, even with recent advancements in cutting-edge frameworks like YOLOv11 for enhanced tomato and plant disease classification (Eliwa and Abd El-Hafeez, 2025a; Eliwa and Abd El-Hafeez, 2025b). In real-world agricultural environments, a single tomato leaf frequently suffers from concurrent infections by different pathogens, creating a dense and overlapping distribution of symptomatic regions that severely complicates multi-objective identification. Furthermore, the scale of disease manifestations varies drastically, ranging from minute, newly formed punctate lesions in the early stages of infection to expansive, irregular symptomatic areas in later stages. Existing detection models often struggle to balance the high spatial resolution required for capturing tiny local features with the robust semantic information needed for understanding large-area global contexts. This imbalance frequently results in missed detections for early-stage microscopic diseases and false positives in scenarios with severe background interference, underscoring the urgent need for a more robust multi-scale feature matching strategy that can effectively handle these practical pain points.

This study proposes a deployment-oriented tomato leaf disease identification method based on an improved YOLO v8s architecture, covering dataset curation, model design, and performance verification. A high-quality multi-target, multi-scale dataset with complex backgrounds and coexisting lesions using PlantVillage public repositories. Mosaic augmentation, geometric transformations (rotation, flipping, cropping), and photometric adjustments (brightness/contrast) enhance robustness to occlusion, clutter, and small objects by expanding scene and illumination variability. For modeling, YOLO v8s is combined with transfer learning to leverage large-scale pretraining priors, accelerating convergence and mitigating overfitting under limited supervision. A Convolutional Block Attention Module (CBAM) is inserted at the backbone–neck interface to refine channel and spatial features before multi-scale fusion, improving detection of small/low-contrast lesions while maintaining YOLO v8s lightweight property. The method integrates high accuracy, compactness, and real-time inference for resource-constrained devices, providing a practical solution for automated tomato disease monitoring in modern agriculture.

## 2 Materials and methods

### 2.1 Overview

**Figure 1:**
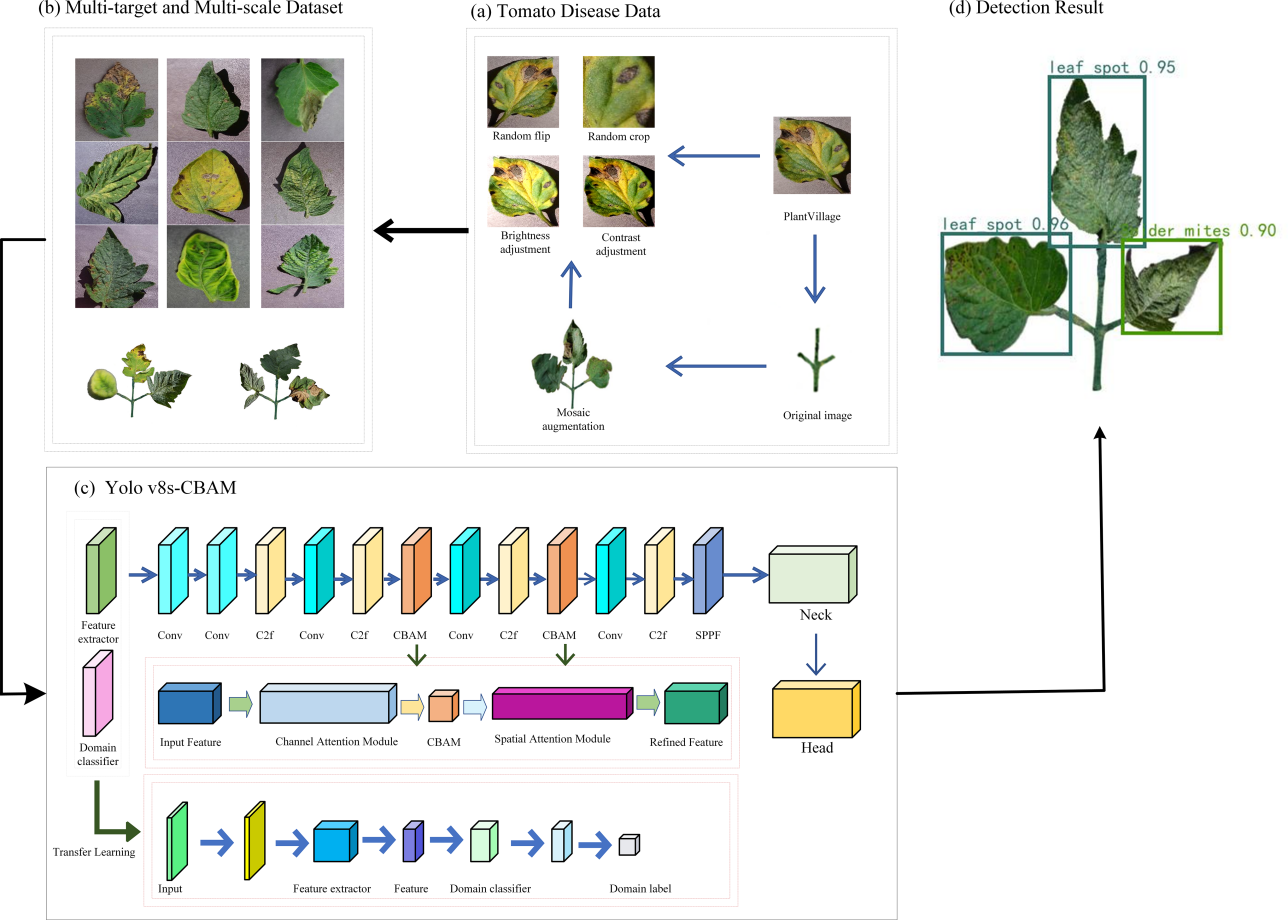
The overall technical pipeline of the YOLOv8s-CBAM. **(a)** Tomato Disease Data: Images from the PlantVillage dataset are subjected to mosaic composition to generate multi-target and multi-scale training samples, supplemented with data preprocessing including geometric transformations and photometric adjustments. **(b)** Multi-target and Multi-scale Dataset: Data balancing is performed on the augmented images to establish a leaf disease dataset with variations in morphology and appearance. **(c)** YOLOv8s-CBAM: The backbone of YOLO v8s is initialized using transfer learning, and the model is further enhanced by inserting CBAM modules to optimize channel-spatial attention. **(d)** Detection Result: The trained model outputs bounding boxes with disease categories and confidence scores, enabling the identification of leaf spots, spider mites, and other disease symptoms.

This study proposes an improved detector, YOLOv8s-CBAM, for multi-target and multi-scale tomato leaf lesion detection. The overall technical pipeline, encompassing data collection and processing, dataset construction, model improvement, and result presentation, is illustrated in **Figure 1**.

Initially, data of nine disease-related categories were downloaded from the public PlantVillage dataset, followed by the synthesis of complex multi-target and multi-scale disease data. Subsequently, data augmentation was performed, as shown in **Figure 1a**. A multi-target and multi-scale dataset was then established (**Figure 1b**). In addition to these pathogenic categories, the dataset includes one “Healthy” class and one synthetically generated “Multi-Target Disease” class. Each of the nine categories contains exactly 1, 000 images, ensuring a balanced distribution to facilitate effective model training. In terms of model design, transfer learning was adopted to initialize the backbone of YOLO v8s with pre-trained weights, aiming to accelerate model convergence and alleviate overfitting. Meanwhile, the CBAM was inserted at the backbone–neck interface to enhance channel-spatial attention, which effectively improves the localization stability of small lesions (**Figure 1c**). Eventually, a multi-target and multi-scale tomato leaf disease detection result was obtained, as presented in **Figure 1d**.

### 2.2 Dataset construction

#### 2.2.1 Multi-target synthesis

This study employs a “cutout-and-paste” mosaic composition method to construct multi-target and multi-scale tomato leaf disease samples, aiming to simulate real-world field scenarios characterized by dense targets, cross-scale lesions, and complex backgrounds (Li et al., 2022). Specifically, 375 images are randomly sampled from each category and grouped into triplets to generate 1, 000 composite images. For each composite image, the three source images are first randomly resized within the range of 0.5–1.5× their original size with a ±10% aspect-ratio jitter, and then placed on a shared canvas at random offsets defined in (Equation 1), allowing partial overlap. This process generates dense, cross-scale scenes with coexisting lesions and cluttered backgrounds, which not only enhances the model’s robustness to small targets, overlapping targets, and texture-blurred regions but also avoids redundancy with the subsequent data augmentation section.

(1) $$I_{\text{composite}}=\overset{3}{\underset{k=1}{\cup }}T(I_{k};s_{k},r_{k},p_{k}),\;\text{s.t.}\{\begin{array}{l} s_{k}\sim \text{U}(0.5,1.5),\\ {}[3pt]r_{k}\sim \text{U}(0.9,1.1),\\ {}[3pt]p_{k}=(x_{k},y_{k})\in \Omega . \end{array}$$

where T denotes the affine transformation function applied to the *k*-th source image *I_k_*. The parameters *s_k_* and *r_k_* represent the scaling factor and aspect ratio, sampled from uniform distributions U(0.5,1.5) and U(0.9,1.1), respectively. The coordinate vector *p_k_* = (*x_k_*, *y_k_*) determines the placement of the image within the shared canvas domain Ω, allowing for random offsets and partial overlap.

The generation of target bounding boxes and category labels for the synthesized Mosaic images is performed through an automated coordinate mapping algorithm based on the geometric transformation matrix. For each of the four randomly selected sub-images, their original ground-truth coordinates are rescaled and translated according to their respective positions in the $640 \times 640$ mosaic canvas. To address the issue of overlapping and truncated lesions at the stitching boundaries, we implement a threshold-based filtering strategy: any bounding box that is truncated to less than 20% of its original area or whose aspect ratio becomes distorted beyond physical plausibility is automatically discarded to prevent the introduction of noise. For overlapping regions, the labels are maintained in a multi-layer format to ensure the model learns to decouple partially occluded symptoms. Furthermore, to verify the accuracy and completeness of these synthesized annotations, a random sample of 5% of the Mosaic-generated images underwent manual visual inspection. This quality control process confirmed that the automated labels remained highly consistent with the visual lesion boundaries, with no significant “label drift” or “phantom targets” observed. This rigorous annotation strategy ensures that the synthetic dataset maintains high fidelity and provides a reliable foundation for training the multi-scale detection heads.

#### 2.2.2 Data augmentation

**Figure 2:**
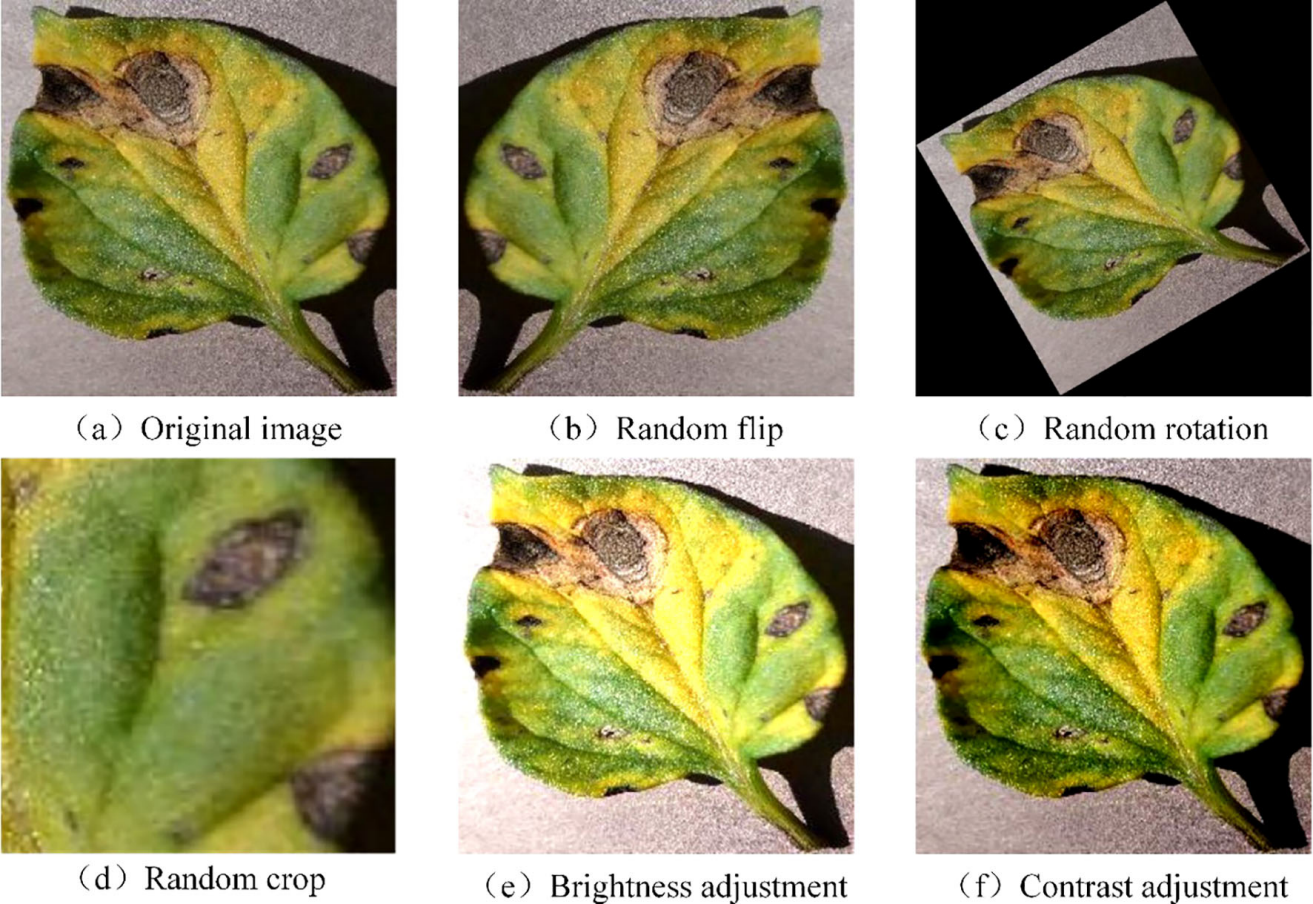
Tomato disease image of data augmentation. **(a)** Original image. **(b)** Random flip. **(c)** Random rotation. **(d)** Random crop; **(e)** Brightness adjustment. **(f)** Contrast adjustment.

To further improve the diversity of the dataset and enhance the model’s generalization capability, a series of data augmentation techniques were applied to the images. Specifically, two primary categories of augmentation methods were adopted (**Figure 2**), with each targeting different sources of variation in field environments. The first category is geometric augmentation, which includes the following operations: random flipping (**Figure 2b**), random rotation (**Figure 2c**), and random cropping (**Figure 2d**). These transformations simulate the diverse perspectives and physical orientations of leaves under natural growth conditions, helping the model break free from its reliance on the specific spatial positions or orientations of lesions. This approach can mitigate the model’s potential bias toward incidental spatial patterns and compel it to focus on the specific characteristics of lesions. The second category is photometric augmentation, which involves random adjustments to image brightness (**Figure 2e**) and contrast (**Figure 2f**). This method simulates the common lighting variations in field environments, which are caused by factors such as weather conditions, light intensity, or time of day, thereby reducing the model’s sensitivity to environmental changes. By replicating these typical lighting variations, the model becomes less dependent on stable illumination and exhibits significantly improved ability to identify diseases under unstable lighting conditions.

Systematic implementation of these two categories of augmentation methods not only effectively increases the sample size and mitigates the class imbalance problem but also, more importantly, enhances the model’s robustness in target recognition under complex and variable real-world scenarios. This rational design ensures that each augmentation operation targets a specific type of real-world variation, thereby collectively improving the model’s generalization capability beyond the original training dataset (Shorten and Khoshgoftaar, 2019). Geometric transformations effectively enhance the model’s invariance to target position, posture, and scale by introducing spatial diversity. Furthermore, as these transformations are essentially pixel-reordering processes, they do not alter the image’s information entropy, texture distribution, or the integrity of lesion features, thereby ensuring the effectiveness and reliability of the augmented images for model training.

#### 2.2.3 Data balancing

**Figure 3:**
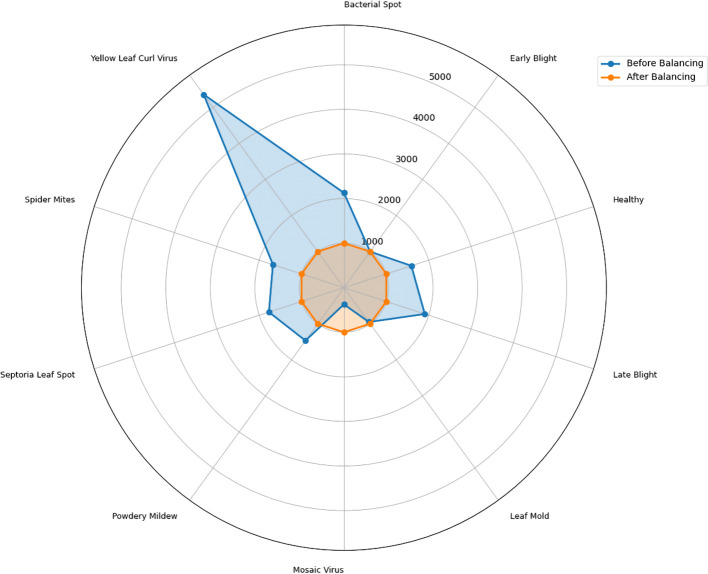
The quantitative distribution before and after data selection and balancing. The initial imbalanced data (orange point) was processed through selection, augmentation, and down-sampling to create the final balanced dataset (blue point).

A class balancing strategy combining oversampling and random down-sampling was employed in this study. It aimed to address the severe class imbalance in the initial ten categories constructed from PlantVillage, thereby preventing the model from being biased toward majority classes during training. As illustrated in **Figure 3**, the number of images varied significantly among the initial categories. To address this, eight selected categories (including seven disease types and one healthy class) were standardized to a uniform size of 1, 000 images. For minority classes, oversampling was utilized to increase the sample count; for majority classes, random down-sampling was performed to select 1, 000 representative images. Finally, by incorporating the synthetically generated “Multi-Target Disease” class, the implementation of this strategy effectively balanced the sample distribution across all nine categories, laying a stable and consistent foundation for subsequent model training. Additionally, geometric data augmentation was applied to the balanced dataset to prevent overfitting.

#### 2.2.4 Dataset characteristics

The entire data processing pipeline began with the public PlantVillage dataset, which contained 18, 161 images across ten initial tomato leaf categories. Following the meticulous curation, balancing, and augmentation processes previously described, the final high-quality dataset for this study was constructed. It now comprises a total of 9, 000 images, equally distributed across nine categories. These include seven selected disease types: Bacterial Spot, Early Blight, Late Blight, Leaf Mold, Septoria Leaf Spot, Spider Mites, and Yellow Leaf Curl Virus. In addition to these diseases, the dataset includes one “Healthy” class and one synthetically generated “Multi-Target Disease” class. Each of these nine categories contains exactly 1, 000 images, ensuring a balanced distribution for model training.

To facilitate model development and evaluation, the dataset was partitioned into training, validation, and test sets. Following a standard 9:1:1 ratio, this resulted in a training set of 7, 290 images for model learning, a validation set of 810 images for hyperparameter optimization, and a test set of 900 images for final performance evaluation. This structured and balanced dataset provides a robust foundation for training and rigorously evaluating the disease detection models in this study. The comprehensive statistics, including the total size and the specific allocation across the training, validation, and test sets for each category, are summarized in **Table 1**.

**Table 1:** Detailed statistical distribution and partitioning of the constructed dataset.

Category name	Total count	Training	Validation	Testing
Bacterial Spot	1, 000	810	90	100
Early Blight	1, 000	810	90	100
Late Blight	1, 000	810	90	100
Leaf Mold	1, 000	810	90	100
Septoria Leaf Spot	1, 000	810	90	100
Spider Mites	1, 000	810	90	100
Yellow Leaf Curl Virus	1, 000	810	90	100
Multi-Target Disease	1, 000	810	90	100
Healthy	1, 000	810	90	100

### 2.3 Convolutional block attention module

**Figure 4:**
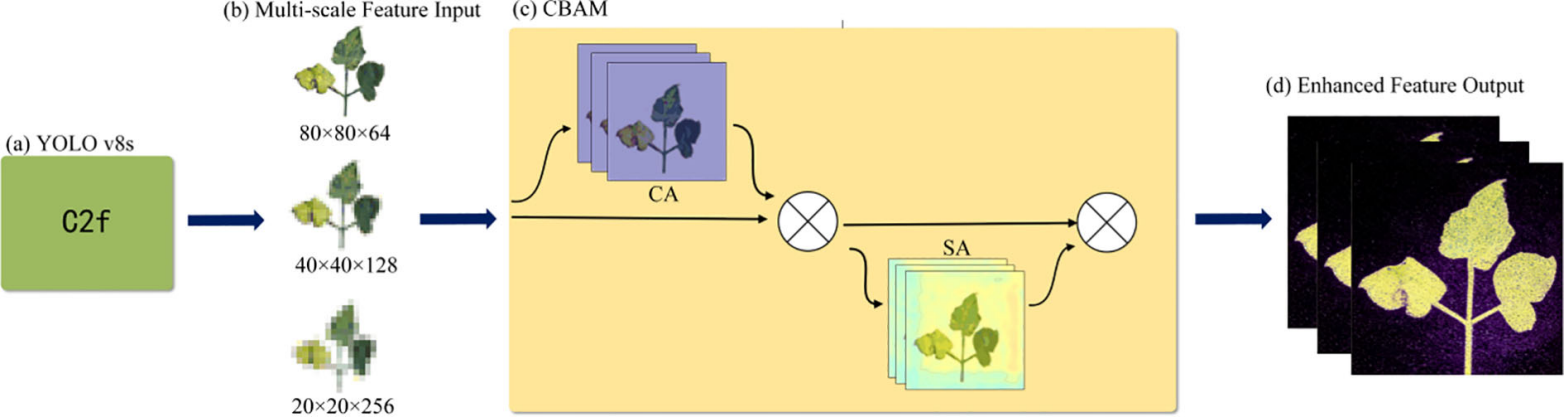
CBAM feature enhancement module for detecting tomato leaf diseases. **(a)** The C2f model of the YOLO v8s. **(b)** Multi-scale feature input. **(c)** CBAM. **(d)** Enhanced feature output.

In this study, the CBAM feature enhancement process is designed to address the core challenges of multi-scale lesion distribution, low contrast between lesions and healthy tissues, and complex background interference, as illustrated in **Figure 4**. Preprocessed tomato leaf images are fed into the backbone network of YOLOv8, and under the YOLO v8s configuration, the C2f module performs cross-stage partial feature fusion and downsampling operations to generate three-scale feature maps: (1) a small-scale 80×80×64 feature map that focuses on capturing early or microscopic lesions while retaining abundant shallow details such as edges and color gradients, (2) a medium-scale 40×40×128 feature map that integrates lesion texture details and preliminary semantic information, and (3) a large-scale 20×20×256 feature map that emphasizes the global distribution characteristics and semantic category information of severe or large-area lesions.

Each scale of feature map is processed by the Convolutional Block Attention Module (CBAM) to achieve accurate feature enhancement and avoid cross-scale interference. For the channel attention (CA) stage, global average pooling and global max pooling are first performed on the input feature maps to obtain two 1×1×C vectors, which are then fed into a shared multi-layer perceptron (MLP) for dimension reduction and activation to generate channel weights (Mc). And for tomato leaf disease detection, the CA module specifically strengthens feature channels related to lesion colors and textures while suppressing redundant channels corresponding to green tissues of healthy leaves and background noise. The feature maps enhanced by channel attention are fed into the spatial attention (SA) stage of CBAM, where average pooling and max pooling along the channel dimension are conducted to generate two H×W×1 feature maps that are concatenated to form an H×W×2 feature map, and a 7×7 convolutional layer is then used for dimension reduction to generate spatial weights (Ms), enabling the SA module to precisely focus on lesion regions and suppress non-lesion structures of leaves and background interference information.

Feature maps enhanced by CBAM, which maintain the same size and number of channels as the input, are output to the neck network PAN-FPN of YOLOv8 for multi-scale feature fusion. The fused features are further fed into the detection head to achieve accurate localization namely bounding box regression and classification of tomato leaf diseases, laying a solid foundation for improving the detection accuracy of multi-scale lesions.

The channel attention module first aggregates channel-wise statistical information using both global average pooling and max pooling, and then generates channel weights through a shared fully connected network to emphasize critical channel features. The channel attention is computed as (Equation 2).

(2) $$\begin{array}{l} M_{c}(F)=\sigma \left(MLP\left(AvgPool(F)\right)+MLP\left(MaxPool(F)\right)\right)\\ \;=\sigma \left(W_{1}\left(W_{0}F_{\text{avg}}^{c}\right)+W_{1}\left(W_{0}F_{\text{max}}^{c}\right)\right) \end{array}$$

Where σ denotes the sigmoid function, **W**_0_ and **W**_1_ represent the weights of the shared *MLP*, and $F_{\text{avg}}^{c}$ and $F_{\text{max}}^{c}$ denote the average-pooled and max-pooled features, respectively.

Subsequently, the spatial attention module models the importance of spatial locations on the weighted feature maps using convolution, guiding the model to focus on target regions. The spatial attention is computed as (Equation 3).

(3) $$M_{s}(F)=\sigma \left(f^{7\times 7}\left(\left[AvgPool(F);MaxPool(F)\right]\right)\right)=\sigma \left(f^{7\times 7}\left(\left[F_{\text{avg}}^{s};F_{\text{max}}^{s}\right]\right)\right)$$

where σ denotes the sigmoid function, *f*^7×7^ represents a convolution operation with a filter size of 7×7, and $\left[F_{\text{avg}}^{s};\;[cite_{\text{s}}tart]\;F_{\text{max}}^{s}\right]$ denotes the concatenation of the average-pooled and max-pooled features across the channel axis.

The sequential application of these two modules allows the model to effectively extract highly discriminative features associated with diseases, even in complex backgrounds, thereby improving the detection accuracy for multi-target and multi-scale lesions.

### 2.4 The YOLOv8s-CBAM network

**Figure 5:**
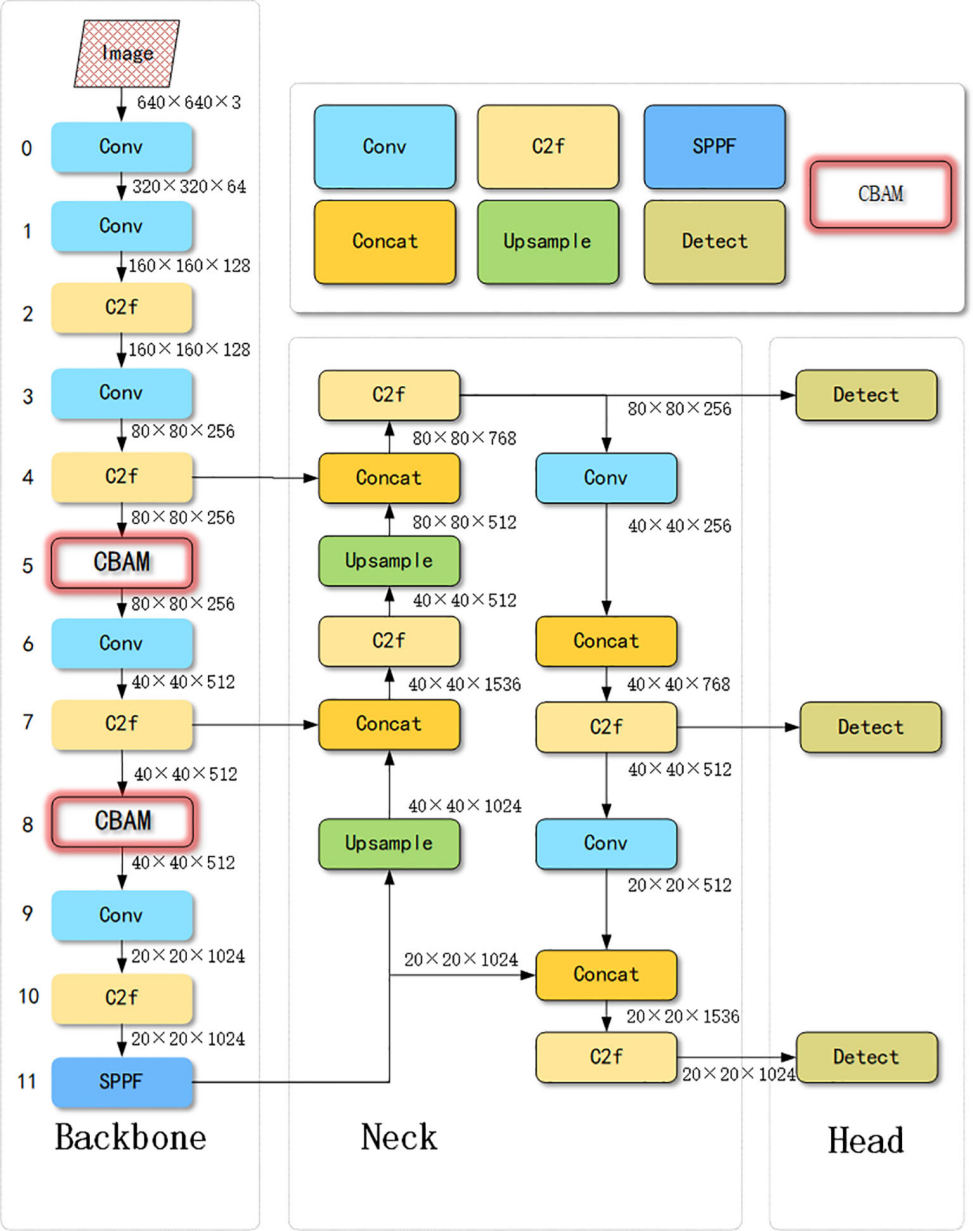
Architecture of the improved YOLO v8s incorporating the CBAM module. Two CBAM modules are respectively inserted after the two C2f modules in the backbone network.

This study proposes an improved strategy for the YOLO v8s model by the CBAM attention mechanism to optimize the original architecture. The CBAM attention mechanism was further integrated into the YOLO v8s structure to strengthen the model’s capacity to focus on critical feature regions. Specifically, the CBAM module was embedded into the P3/8 and P4/16 feature extraction layers of the Backbone. This placement was strategic: the P3/8 feature map, with its 80x80 resolution, offers higher resolution, enabling the capture of fine-grained details and small targets, such as the small lesions characteristic of Bacterial Spot and Septoria Leaf Spot. Conversely, the P4/16 feature map, which has a 40x40 resolution, provides stronger semantic information, making it more effective for extracting the overall features of larger disease areas, such as the complex patterns of Yellow Leaf Curl Virus. By embedding CBAM at these key multi-scale stages, the model can dynamically adjust attention across both channel and spatial dimensions. The channel attention module models the response intensity along the channel dimension, amplifying feature channels that are highly relevant to disease lesions. Subsequently, the spatial attention module generates a two-dimensional spatial attention map, guiding the model to focus on potential lesion locations within the image. The sequential application of these two modules allows the model to effectively extract highly discriminative features associated with diseases, even in complex backgrounds, thereby improving the detection accuracy for multi-target and multi-scale lesions. **Figure 5** presents an overview of the improved YOLO v8s model architecture, indicating the integration sites of the CBAM modules.

### 2.5 Evaluation metric

In this study, four evaluation metrics—Precision, Recall, F1-Score, and Mean Average Precision (mAP)—were adopted to comprehensively assess the detection performance of the model in the task of tomato leaf disease identification, considering classification accuracy, detection completeness, overall performance, and multi-class detection capability.

**Precision:** Precision measures the proportion of true positive predictions among all samples predicted as positive, as defined in (Equation 4), where TP denotes the number of true positives and FP denotes the number of false positives. A higher precision indicates fewer false alarms and more accurate prediction results.

(4) $$\text{Precision}=\frac{TP}{TP+FP}$$

**Recall:** Recall represents the proportion of correctly identified positive samples among all actual positive samples, as shown in (Equation 5). Here, FN denotes the number of false negatives. A higher recall indicates a more comprehensive detection ability and a lower missed detection rate.

(5) $$\text{Recall}=\frac{TP}{TP+FN}$$

**F1-Score:** The F1-Score is the weighted harmonic mean of precision and recall, providing a comprehensive evaluation of model performance, as described in (Equation 6). Values closer to 1 indicate that the model achieves a better balance between precision and recall.

(6) $$F1=2\times \frac{\text{Precision}\times \text{Recall}}{\text{Precision}+\text{Recall}}$$

**Mean Average Precision (mAP):** Mean Average Precision evaluates the overall detection capability of the model across all target categories and is a core metric in object detection tasks, as defined in (Equation 7). For each class, the area under the Precision-Recall curve at different confidence thresholds is calculated as the Average Precision (AP), and the mean value across all classes is taken as mAP. mAP can be computed at different Intersection over Union (IoU) thresholds; mAP@0.5 denotes mAP at an IoU threshold of 0.5, while mAP@0.95 refers to the average mAP across IoU thresholds from 0.5 to 0.95, which is currently the most widely used comprehensive metric in object detection. A higher mAP indicates more balanced and superior detection capability across all categories.

(7) $$\text{mAP}=\frac{\sum_{\text{i}=1}^{N}AP_{i}}{N}$$

## 3 Results

### 3.1 Detailed settings

The experimental environment in this study was established on a local training platform that balances performance and cost, suitable for the development and training of small- to medium-scale object detection tasks. In terms of hardware, an AMD Ryzen 5–3600 six-core processor was used for data preprocessing and loading, paired with an NVIDIA GeForce GTX 1650 SUPER graphics card supporting CUDA acceleration. Although the GPU has limited memory, it provides adequate training performance for moderate-scale tasks. A total of 36 GB RAM ensures smooth operation during large-scale data loading. For software, the system operates on Windows 10, with Python 3.8 and PyTorch 2.2.1 used to construct the training environment. cuDNN 12.1 was incorporated to enable GPU acceleration, and PyCharm served as the development tool, offering efficient code management and debugging capabilities. During model training, to ensure fairness and reproducibility, all object detection models were configured with the same hyperparameters. According to the characteristics of the dataset and GPU memory constraints, the batch size was set to 4, allowing stable training while preventing memory overflow. The number of training epochs was set to 20, ensuring sufficient learning of data features. The input image size was set to 640×640, balancing recognition accuracy and computational cost. This configuration demonstrated stable training and good resource adaptability in the local environment. The detailed configuration is presented in **Table 2**.

**Table 2:** Hardware configuration and operating environment.

Configuration item	Parameter
*CPU*	AMD Ryzen 5 3600, 6-Core
*GPU*	NVIDIA GeForce GTX 1650 SUPER
*Memory*	Asgard DDR4 3200MHz 8GB x 4
*Python*	3.8
*Pytorch*	2.2.1
*cuDNN*	12.1
*Batch size*	4
*Epochs*	20
*Input image size*	640×640

### 3.2 Visualization of the training process

**Figure 6:**
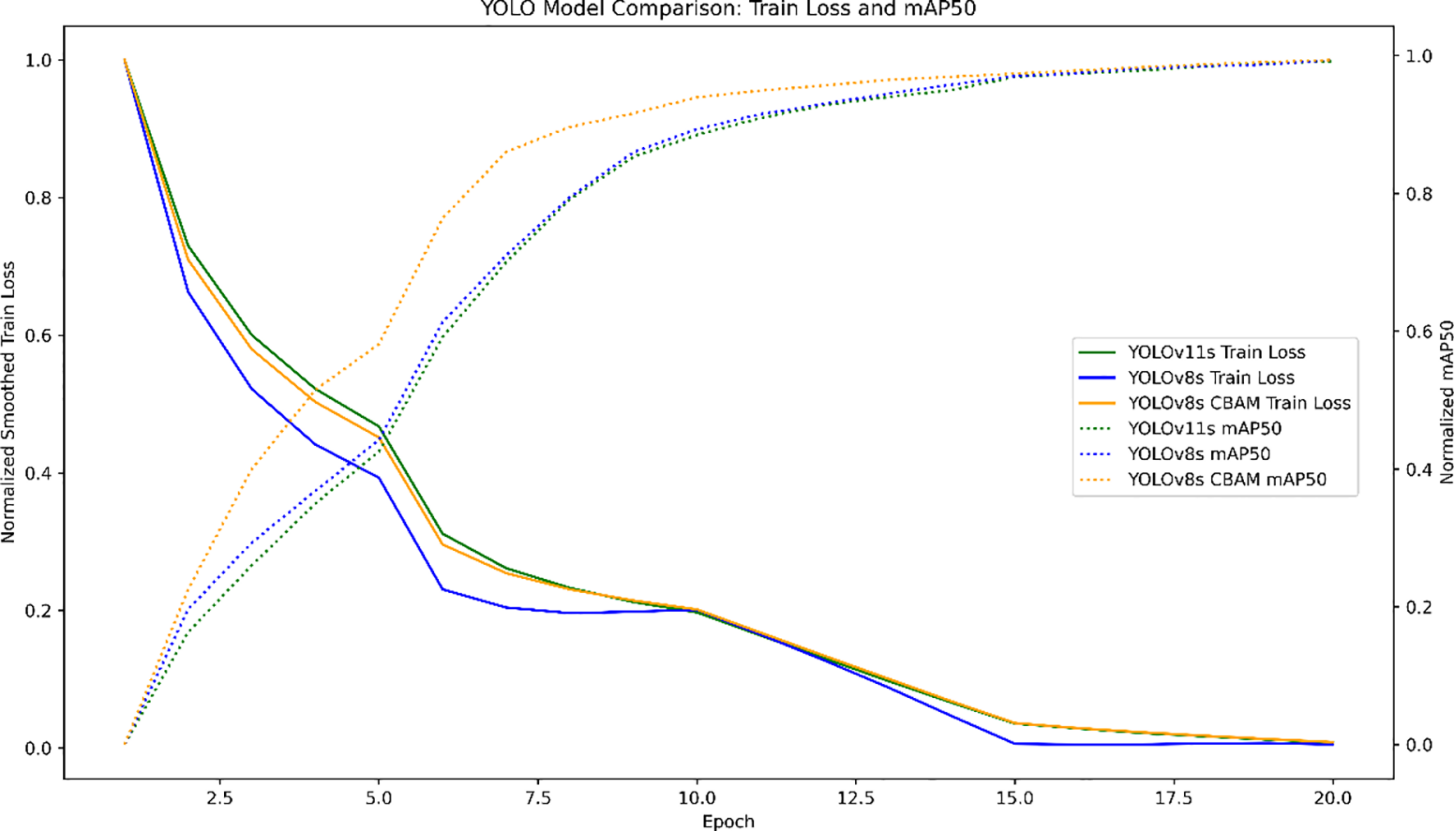
Comparison of different methods for train loss and mAP50. For all models trained with the same schedule and number of epochs, the results of Loss and mAP@0.5 demonstrate that YOLOv8s-CBAM converges more rapidly and attains a superior stable accuracy.

To assess the influence of the attention mechanism on the model’s training performance, this study compared the descending trends of the loss function and variations in the mAP@0.5 metric across three models: YOLOv8s-CBAM, YOLO v8s, and YOLOv11s, with the results presented in **Figure 6**. With the increase in epochs, the YOLOv8s-CBAM model exhibited clear convergence around the fifth epoch, with the loss value rapidly decreasing and stabilizing, achieving a convergence speed approximately 20% faster than that of YOLO v8s. Simultaneously, the mAP@0.5 curve of YOLOv8s-CBAM quickly rose to 0.99 within the first five training epochs and remained stable in subsequent epochs, representing an improvement of about 2.4% over YOLO v8s and 4.1% over YOLO v11s.In comparison, although YOLO v8s ultimately achieved favorable accuracy, its initial convergence was slower, with a more gradual mAP improvement. YOLO v11s demonstrated a certain advantage in loss reduction speed, yet its detection accuracy was significantly lower than that of the other two models. Analysis of the training loss and mAP curves indicates that YOLOv8s-CBAM demonstrates stronger feature learning and discrimination capabilities in the early stages of training. This suggests that the CBAM attention module guides the model to focus on lesion-critical regions, accelerating training and improving generalization. Overall, the YOLOv8s-CBAM model outperforms the other models in terms of training efficiency, convergence speed, and final detection performance, verifying the synergistic enhancement effect of the attention mechanism and transfer learning.

### 3.3 Visualization of lesion feature maps

**Figure 7:**
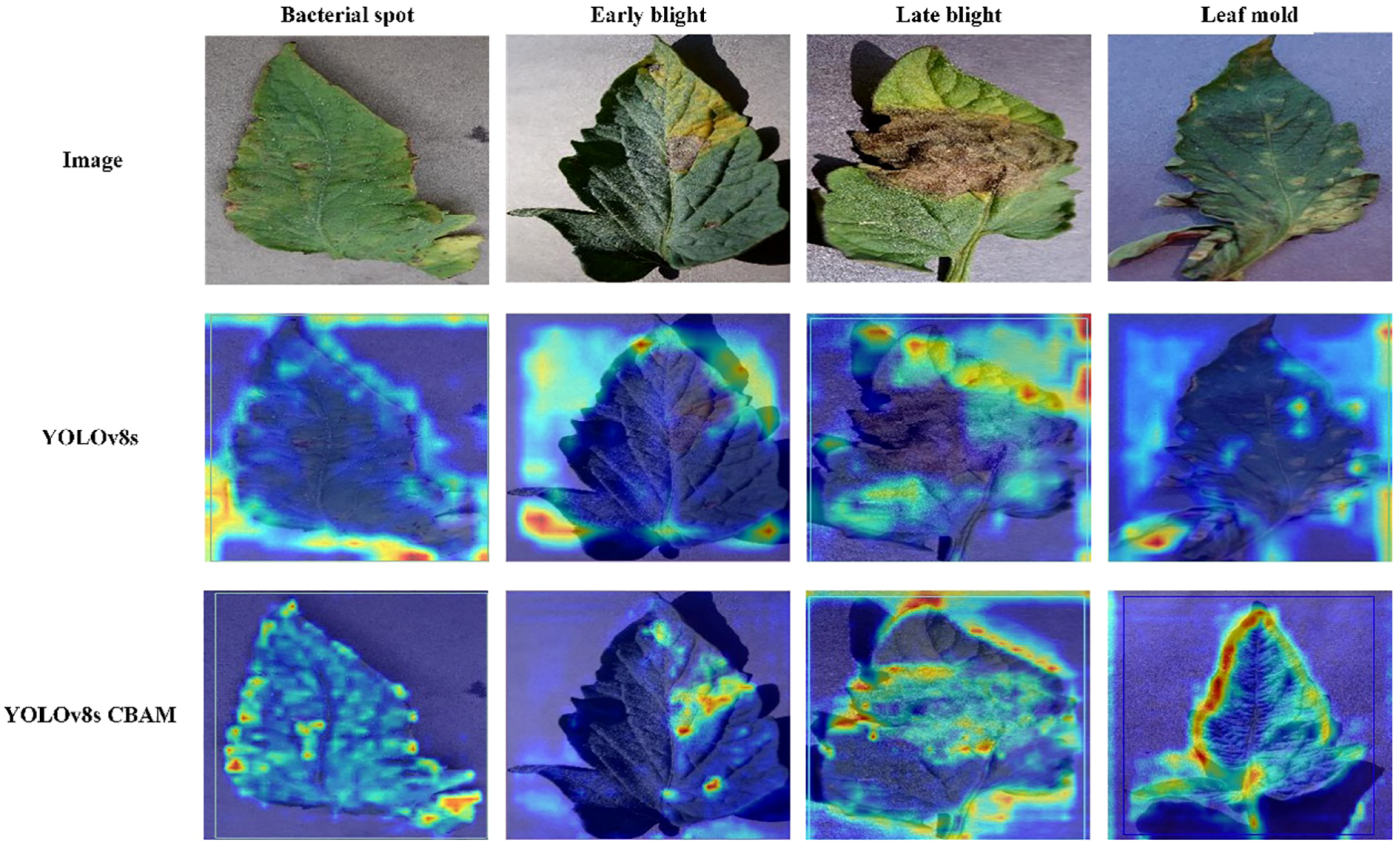
Grad-CAM visualization targeting four tomato leaf disease types. Heatmaps show that YOLOv8s-CBAM concentrates on true lesion areas and suppresses background clutter, while the baseline spreads attention. Where red/yellow regions denote the model’s high-response feature focus areas.

**Figure 8:**
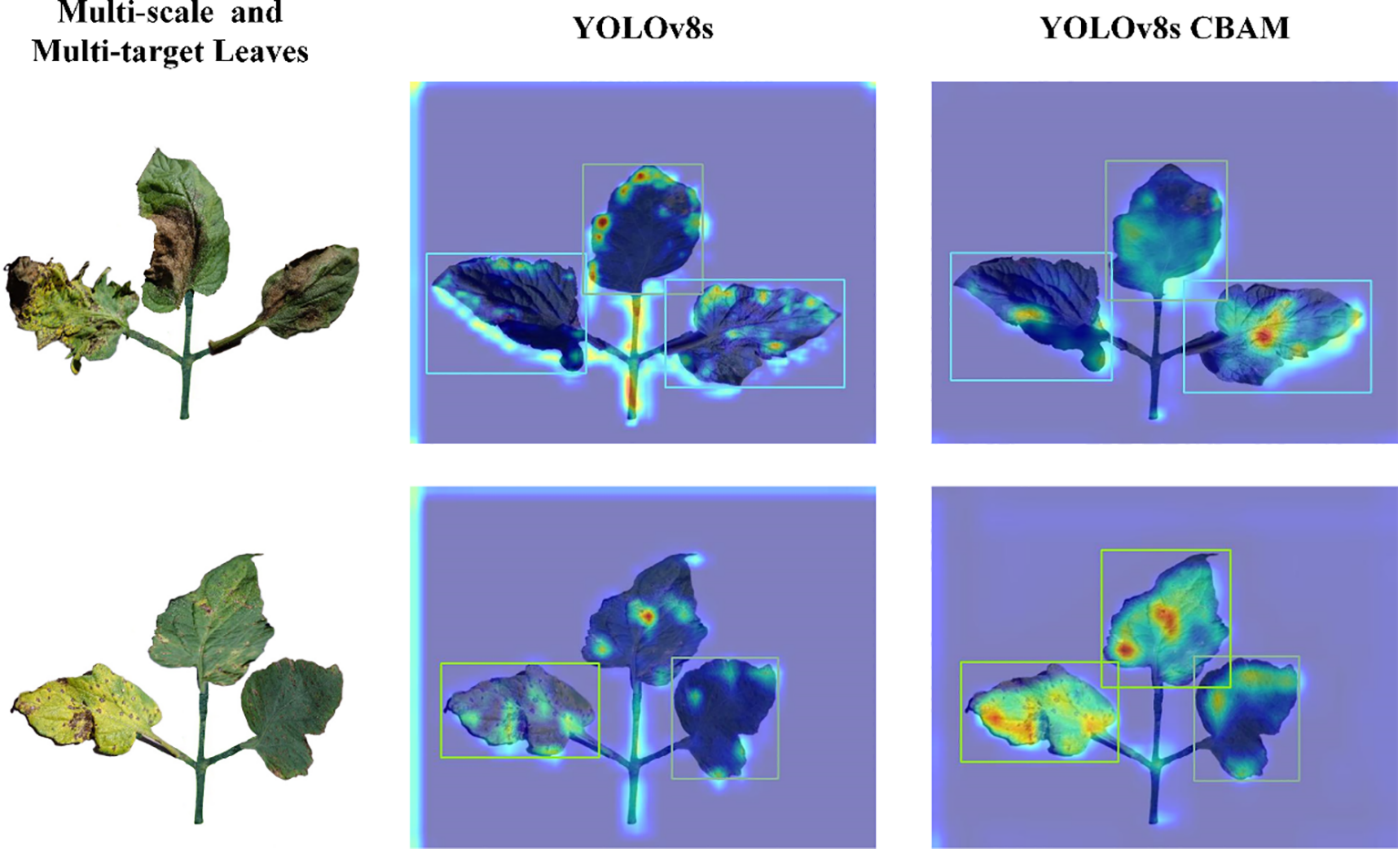
Grad-CAM visualization targeting Multi-Target and Multi-Leaf Tomato Disease leaves. Heatmaps show that YOLOv8s-CBAM concentrates on true lesion areas and suppresses background clutter, while the baseline spreads attention. Where red/yellow regions denote the model’s high-response feature focus areas.

**Figure 9:**
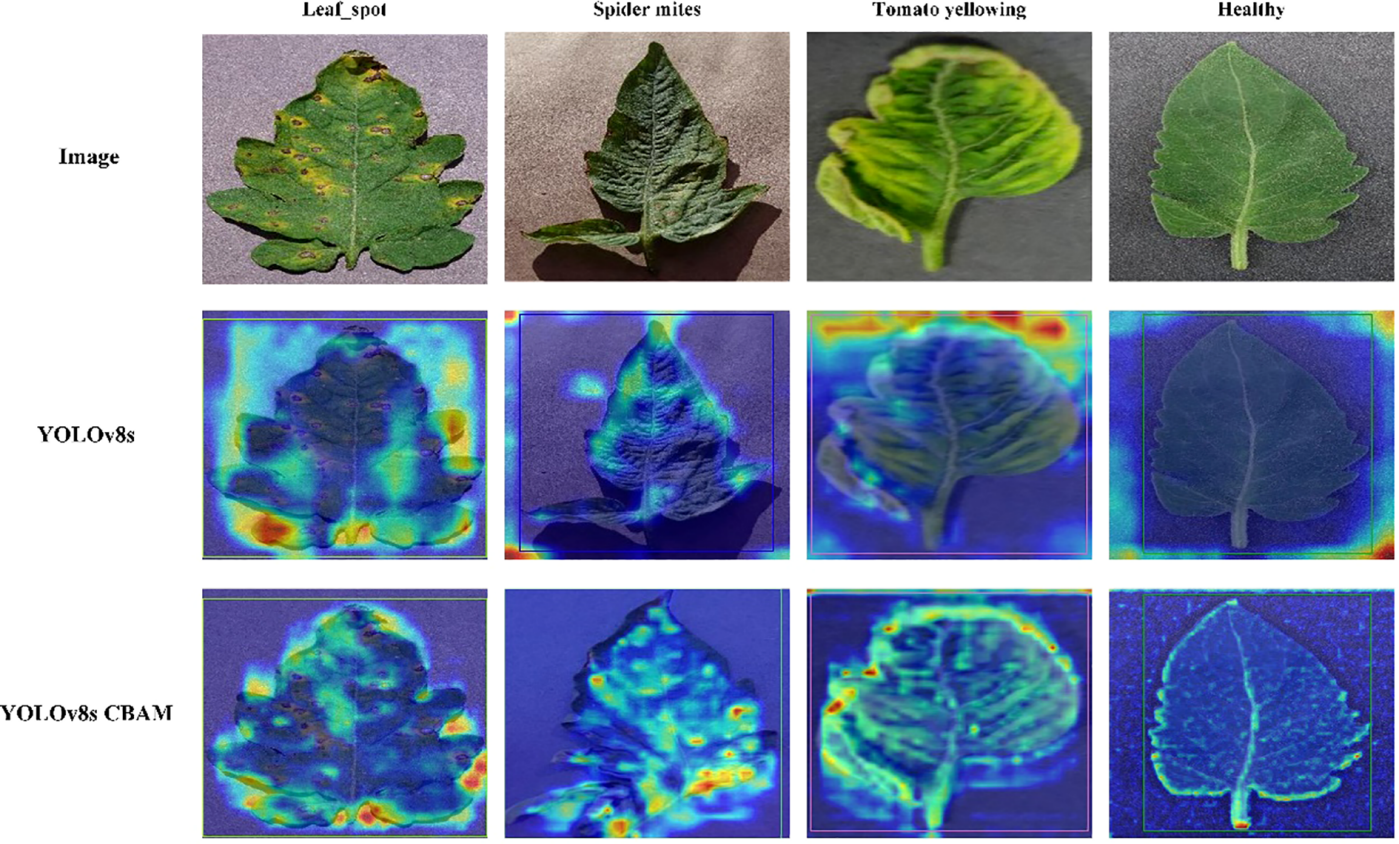
Grad-CAM visualization targeting three tomato leaf disease types and healthy leaf. Heatmaps show that YOLOv8s-CBAM concentrates on true lesion areas and suppresses background clutter, while the baseline spreads attention. Where red/yellow regions denote the model’s high-response feature focus areas.

To provide a more intuitive analysis of the regions of interest for deep learning models in tomato leaf disease detection tasks, this study employed Gradient-weighted Class Activation Mapping (Grad-CAM) to visualize the model’s decision-making process. **Figures 7****–9** present the Grad-CAM visualizations of both the original YOLO v8s model and the improved model incorporating the CBAM module on the same disease images. The results indicate that the original YOLO v8s model exhibits significant background interference during recognition, with dispersed attention and frequent misallocation of focus to non-lesion regions in the heatmaps, resulting in insufficient ability to concentrate on the target lesions.

#### 3.3.1 Feature attention mapping for disease categories (group I)

**Figure 7** illustrates a comparative analysis of attention heatmaps for four typical tomato disease samples (Bacterial spot, Early blight, Late blight, and Leaf mold), generated by the baseline YOLO v8s model and the improved YOLOv8s-CBAM model. For Bacterial spot characterized by subtle and punctate lesions, YOLO v8s exhibits scattered attention regions with weak response signals, failing to precisely capture the discrete lesion sites. In contrast, YOLOv8s-CBAM accurately identifies the punctate damaged textures and concentrates high-intensity attention on each small lesion area, significantly enhancing the capture of fine-grained disease features. For Early blight marked by concentric ring-shaped lesions, YOLO v8s shows diffused attention responses across the leaf, with poor discrimination between lesion boundaries and healthy tissues. Conversely, YOLOv8s-CBAM centrally targets the core ring-shaped lesion regions, strengthening the spatial localization of disease-specific morphological features. For Late blight characterized by mold-covered large-area lesions, YOLO v8s demonstrates weak response intensity in the dense mold regions, with incomplete coverage of the actual diseased areas. In contrast, YOLOv8s-CBAM’s high-response1 regions fully overlap with the mold-covered lesion areas, effectively capturing the large-scale diseased features. For Leaf mold with yellowish distorted lesion areas, YOLO v8s exhibits scattered attention distribution, failing to align with the distorted lesion boundaries. Conversely, YOLOv8s-CBAM precisely focuses on the yellowish distorted regions, improving the capture of morphology-altered disease features. Notably, by incorporating the CBAM attention module, YOLOv8s-CBAM demonstrates superior performance in capturing diverse lesion features (from fine-grained punctate spots to large-area mold-covered regions), effectively mitigating the limitations of the baseline YOLO v8s in lesion localization and feature discrimination, and thus substantially improving the accuracy and robustness of tomato disease detection.

#### 3.3.2 Feature attention mapping for disease categories (group II)

**Figure 9** illustrates a comparative analysis of attention heatmaps for three tomato leaf disease categories as well as healthy leaf samples, generated by the baseline YOLO v8s model and the improved YOLOv8s-CBAM model. For leaf spot samples, YOLO v8s only induces weak responses in partial lesion areas, whereas YOLOv8s-CBAM enables precise and high-intensity coverage of spotted lesion regions. For subtle lesions, YOLO v8s displays scattered attention regions with weak response signals, while YOLOv8s-CBAM accurately captures the damaged textures of leaves and concentrates on the actual infested sites. For leaf yellowing samples featuring large-area lesions, YOLO v8s exhibits low discriminative ability between healthy and yellowed regions, whereas YOLOv8s-CBAM centrally responds to the core yellowing areas, thereby enhancing the discriminative power of features for non-spotted diseases. Even for healthy leaves, YOLO v8s exhibits invalid attention with locally abnormal high responses, while YOLOv8s-CBAM demonstrates more homogeneous response patterns, effectively mitigating false attention. Notably, by incorporating the CBAM attention module, YOLOv8s-CBAM not only enhances targeted attention toward typical diseases and improves the capture of subtle lesion features but also boosts the discriminability between diseased and healthy samples. This optimization significantly elevates the accuracy and robustness of tomato leaf disease detection at the level of feature attention allocation.

**Figure 8** illustrates a comparative analysis of attention heatmaps for two multi-target multi-leaf tomato disease samples, each consisting of multiple diseased leaves, generated by the baseline YOLO v8s model and the improved YOLOv8s-CBAM model. For the first sample characterized by multiple leaves with local lesions, YOLO v8s exhibits scattered attention regions and weak response signals, and its bounding boxes fail to align accurately with actual lesion sites. In contrast, YOLOv8s-CBAM can precisely identify the damaged textures of each leaf, concentrate high-intensity attention on real lesion areas, and effectively suppress invalid attention to healthy regions, thereby achieving accurate localization of multi-target lesions. For the second sample characterized by leaves with large-area yellowing and spotted lesions, YOLO v8s shows weak response intensity in large-area lesion regions, and its bounding boxes miss part of the lesions. Conversely, the high-response regions of YOLOv8s-CBAM fully cover all yellowing and spotted lesions, significantly enhancing the capability of capturing multi-scale lesion features. Notably, by incorporating the CBAM attention module, YOLOv8s-CBAM demonstrates superior performance in multi-target and multi-scale lesion detection scenarios, effectively mitigating the limitations of the baseline model and substantially improving the accuracy and robustness of multi-leaf tomato disease detection.

### 3.4 Multi-target and multi-scale tomato disease detection

#### 3.4.1 Multi-scenario tomato disease detection

**Figure 10:**
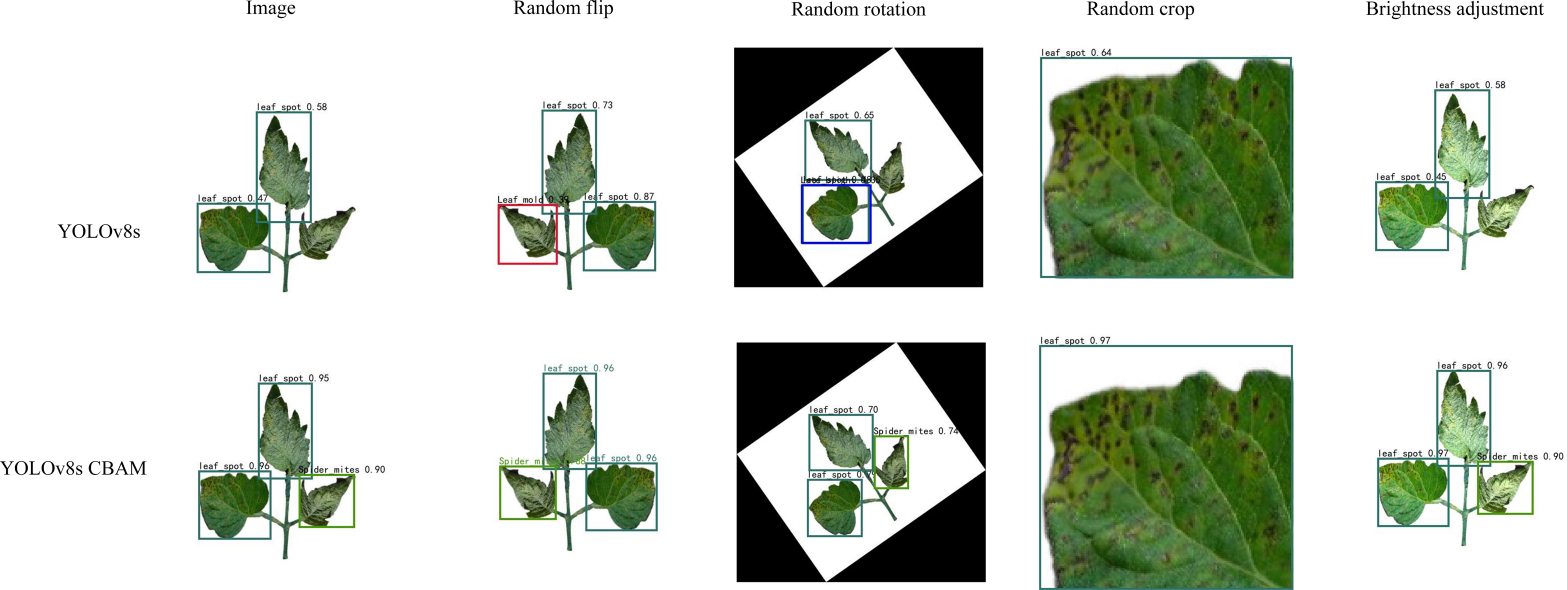
Robustness under perturbations. Qualitative results on flipping, rotation, cropping and brightness changes. YOLOv8s-CBAM maintains reliable labels and confidence in multi-target scenes.

To address the core challenge of multi-scale and multi-target leaf detection, this study constructed diversified test scenarios involving random flipping, rotation, cropping, and brightness adjustment. A comparative analysis of the detection performance between the YOLO v8s model and the improved YOLOv8s-CBAM model was further conducted, and the results are presented in **Figure 10**. In the original image test, the YOLO v8s model only detected leaf spot disease with confidence scores of 0.58 and 0.47 for two lesion areas, respectively, and there were obvious missing detections. In contrast, the YOLOv8s-CBAM model increased the detection confidence score of leaf spot disease to 0.96 and successfully identified spider mite damage (confidence score of 0.90) at the same time, achieving more comprehensive disease coverage detection. In the random flipping and rotation scenarios, the confidence scores of the YOLO v8s model for leaf mold were only 0.73 and 0.87, and it misclassified spider mite damage as leaf mold. However, the YOLOv8s-CBAM model maintained a high confidence score of 0.96 for leaf spot disease consistently and could accurately identify spider mite damage. Notably, in the rotation scenario, the YOLO v8s model performed poorly, exhibiting problems of duplicate annotations and low confidence scores. In contrast, the YOLOv8s-CBAM model maintained the detection confidence score of leaf spot disease above 0.70, could accurately locate multiple disease areas, and showed superior robustness and adaptability. In the task of tomato leaf disease identification, there are significant differences in the size, shape, and distribution of lesions, which makes multi-target and multi-scale recognition a key factor in improving model performance.

#### 3.4.2 Multi-scale and multi-target tomato disease detection

We compared it against several mainstream detectors to verify the overall performance advantages of the improved model. These detectors include YOLO v8s, YOLO v5s, Faster R-CNN, and SSD. The evaluation was conducted using five key metrics: precision, recall, F1-score, mAP@0.5, and inference speed. Detailed comparative results are summarized in **Table 3**. YOLOv8s-CBAM delivered strong results across all metrics. The model reached mAP@0.5 of 0.991 and F1-score of 0.970 and matched the baseline YOLO v8s on headline accuracy while exhibiting higher stability across categories and scenes. YOLO v8s achieved the best single-metric precision at 0.975, yet its performance varied more noticeably between disease types, which reduced consistency in multi-target recognition. By introducing attention, YOLOv8s-CBAM maintained high overall accuracy and improved robustness and generalization in complex scenes with coexisting lesions and small targets, reducing sensitivity to background clutter and illumination changes. Faster R-CNN attained mAP@0.5 of 0.961 and demonstrated solid localization, but its deep two-stage architecture and large parameter count produced a 108 MB model and a precision of 0.817, which implies more false positives and weaker suitability for real-time, lightweight deployment. SSD lagged on every metric, with recall at 0.432 and F1-score at 0.566, indicating frequent missed detections for tomato leaf diseases. The shortfall stems from limited feature fusion and insufficient sensitivity to fine-scale lesions. Overall, YOLOv8s-CBAM provides the best balance of accuracy, efficiency, and deployment readiness, offering reliable performance for multi-target, multi-scale tomato disease detection on edge devices.

**Table 3:** Detection results with different disease detection networks.

Model	Precision	Recall	mAP50	F1 Score	Model size(MB)
SSD	0.887	0.432	0.813	0.566	95.1
Faster R-CNN	0.817	0.943	0.961	0.875	108
YOLO v11s	0.936	0.931	0.979	0.930	**18.7**
YOLO v8s	**0.975**	0.964	0.986	0.970	21.9
YOLO v8s CBAM	0.969	**0.973**	**0.991**	**0.970**	24.8

Performance metrics including Precision, Recall, F1-score, and mAP@0.5 are reported, alongside model size for comprehensive assessment.

Bold values indicate the best performance for each metric.

#### 3.4.3 Different tomato disease detection

**Figure 11:**
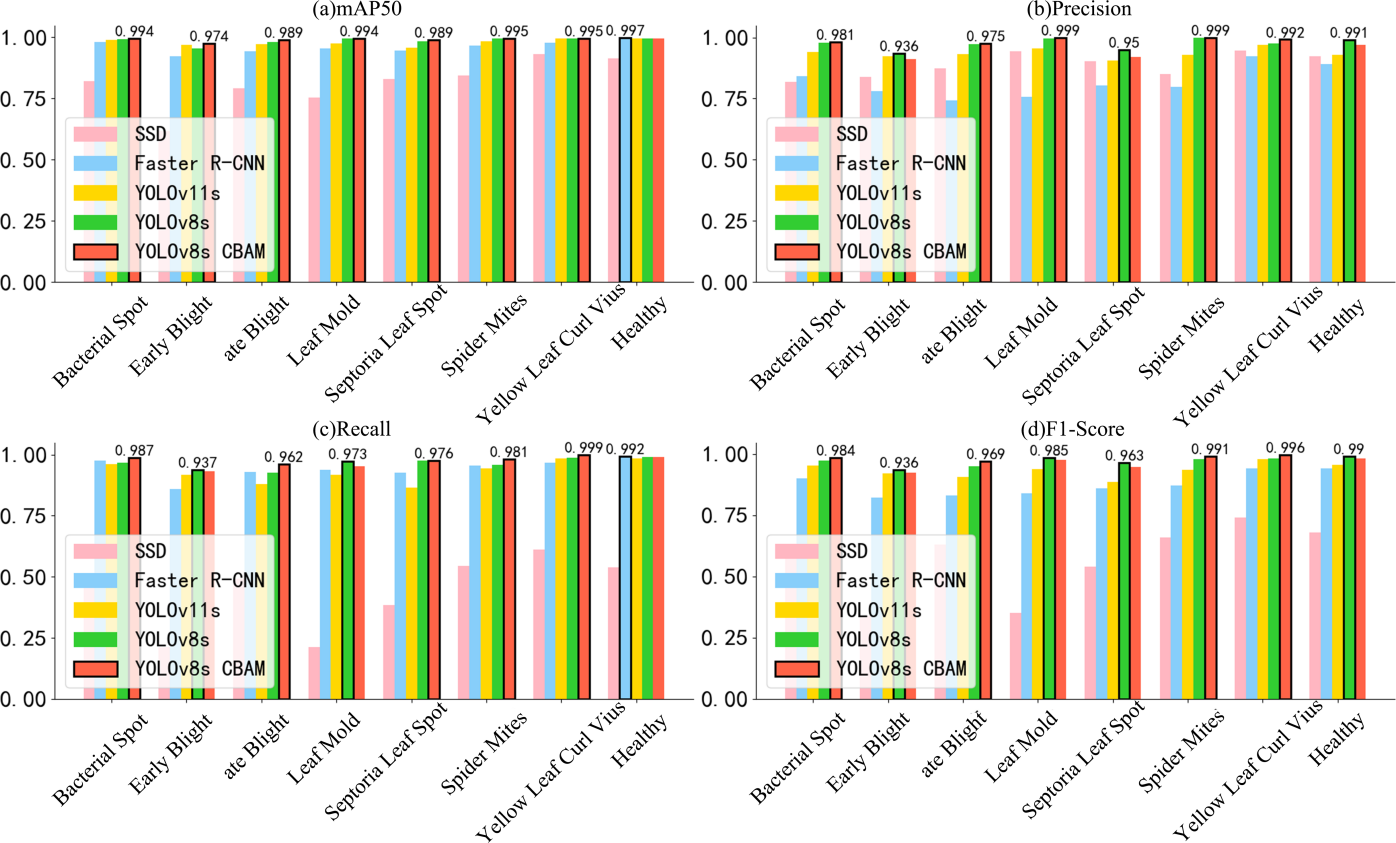
Per-class metrics of different disease detection networks displayed via bar Charts. **(a)** mAP50. **(b)** Precision. **(c)** Recall. **(d)** F1-Score.

To comprehensively assess the recognition performance of each model across different disease categories, **Figure 11** presents the detailed results for mAP@0.5, Precision, Recall, and F1-score. The results demonstrate that the YOLOv8s-CBAM model exhibits significant advantages across all evaluation metrics, particularly in the detection of small-scale, multi-lesion, and complex background categories, where it shows enhanced robustness and generalization capability. For mAP@0.5, YOLOv8s-CBAM achieved the best results in the most challenging categories—spider mite damage 0.989, leaf mold 0.975, and tomato yellow leaf curl virus 0.999—surpassing the other detectors and indicating superior localization accuracy. YOLO v8s remained competitive overall but trailed slightly on these complex categories, while Faster R-CNN and SSD showed clear weaknesses. In terms of precision, YOLOv8s-CBAM achieved a value exceeding 0.97 for typical diseases including bacterial spot and early blight, while delivering the lowest false-positive rates. This performance demonstrates stronger feature selection capability and fewer spurious detections. Notably, for the spider mite’s category, YOLOv8s-CBAM improved precision by 1.2% compared to YOLO v8s, effectively mitigating the common issue of overlooking small targets. Regarding Recall, YOLOv8s-CBAM achieved recall rates of 0.972 and 0.978 for leaf spot and spider mite damage, respectively, surpassing YOLO v8s and YOLO v11s by approximately 1%–2%. This indicates more comprehensive target coverage and a reduced missed detection rate. For the F1-score, YOLOv8s-CBAM ranked near the top across almost all categories, reaching 0.990 on tomato yellow leaf curl virus and demonstrating high accuracy with strong completeness and balance. Overall, the superior performance of YOLOv8s-CBAM is mainly attributed to the introduction of the CBAM attention mechanism, which significantly enhances the model’s responsiveness to key regions, leading to more effective small target recognition and background suppression. Coupled with transfer learning, this further improves training efficiency and feature generalization, enabling the model to achieve leading performance in multi-class tomato leaf disease recognition tasks. .

### 3.5 Ablation studies

#### 3.5.1 Effectiveness of the transfer learning module

**Figure 12:**
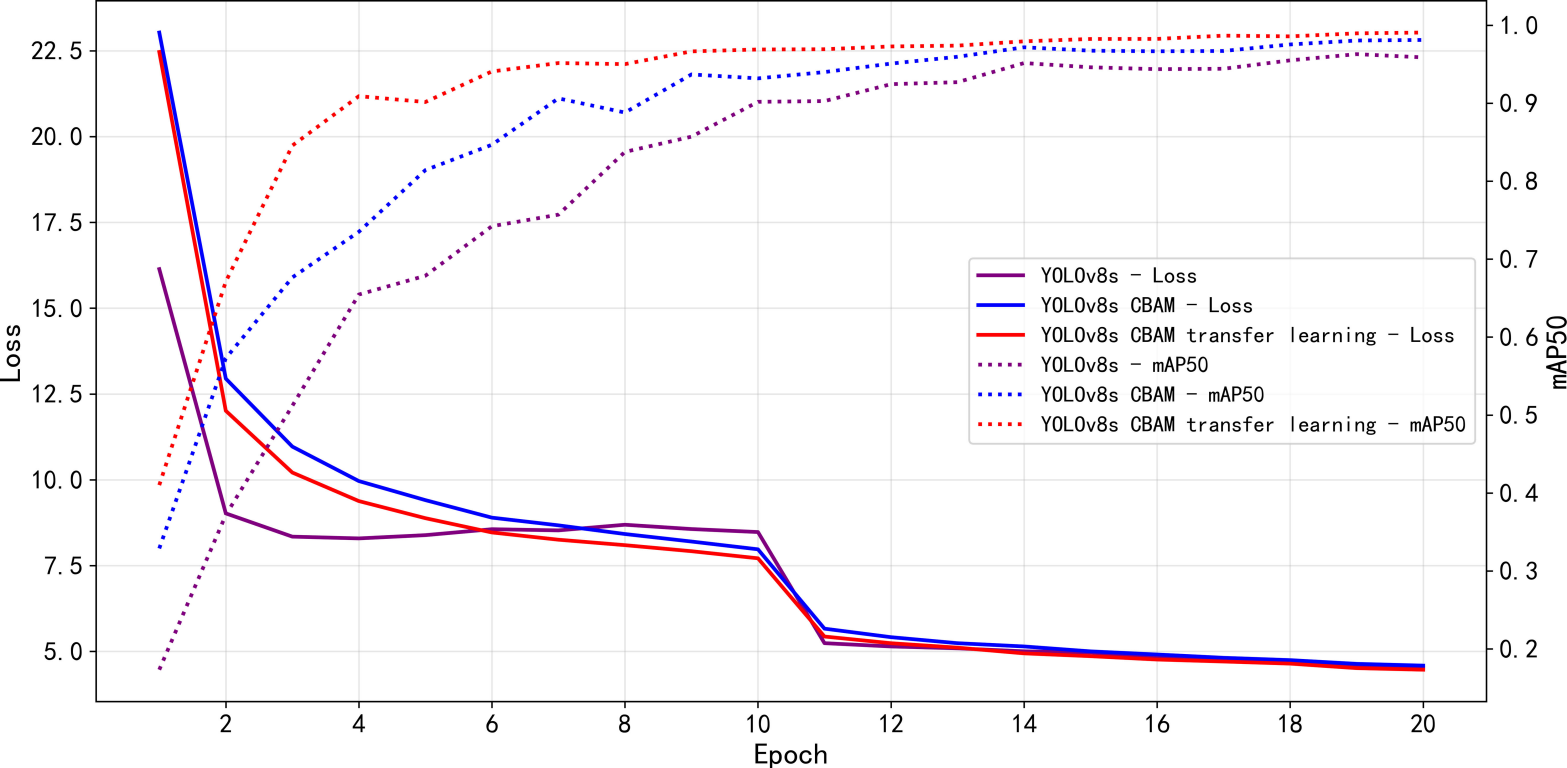
Impact of transfer learning on the YOLOv8s-CBAM architecture. Comparison of training and validation performance with and without transfer learning under a unified improved structure. Solid and dashed lines represent training and validation loss referenced on the left axis. Dotted lines represent mAP50 and mAP50–95 referenced on the right axis. Red and blue colors denote the transfer learning and non-transfer learning variants respectively.

To rigorously evaluate the contribution of transfer learning to the proposed model, we conducted a controlled ablation study using the YOLOv8s-CBAM architecture as a unified baseline. By comparing the model initialized with pretrained weights against the same structure trained from scratch, we analyzed the loss convergence and performance metrics on both the training and validation sets. As illustrated in **Figure 12**, the transfer learning model (YOLOv8s-CBAM-TL) exhibited a significantly more rapid decrease in training loss during the initial stages, achieving near-convergence before the tenth epoch, whereas the model trained from scratch showed a markedly slower descent. On the validation set, the transfer learning approach consistently maintained lower loss values, reflecting its superior generalization capability. Furthermore, the mAP50 metric for the transfer learning model surpassed 0.8 within the first five epochs, while the non-transfer learning counterpart required approximately twelve epochs to reach a comparable level. The higher initial values and faster improvement in mAP50–95 further confirm that the synergy between the CBAM module and transfer learning not only accelerates training efficiency but also substantially enhances detection accuracy under complex multi-scale scenarios.

#### 3.5.2 Effectiveness of the attention mechanism module

Building on this foundation, the SE, GAM, and CBAM modules were individually embedded into either the Backbone or Head of YOLO v8s to evaluate their mAP50 performance across different disease targets. The results in **Table 4** show that the SE module performs well on Early Blight and Septoria Leaf Spot, which are diseases characterized by distinct textures and clear boundaries. The GAM module is more effective on large-area lesions such as Tomato Yellow Leaf Curl Virus and Late Blight, but it falls behind on categories with blurred edges or smaller scales. By contrast, YOLOv8s-CBAM sustains high scores across all categories and excels on small-target or complex-background diseases such as Spider Mites, Leaf Mold, and Early Blight. Its per-class mAP values all exceed 0.99, with an average of 0.994, which clearly surpasses the other models. Overall, the CBAM module, through joint modeling of channel and spatial attention, effectively enhanced the model’s feature focusing and spatial localization abilities. This enables superior generalization and robustness in multi-target and multi-scale disease identification tasks, resulting in optimal overall detection performance and suitability for tomato disease detection in complex, multi-scale environments.

**Table 4:** Comparison of mAP@0.5 for YOLO v8s incorporating different attention mechanisms.

Disease type	YOLO v8s	YOLO v8s+SE	YOLO v8s+CBAM	YOLO v8s+GAM
Bacterial Spot	0.992	0.988	**0.994**	0.984
Early Blight	0.954	0.96	**0.974**	0.967
Late Blight	0.98	0.977	**0.989**	0.967
Leaf Mold	0.993	0.978	**0.994**	0.961
Septoria Leaf Spot	0.985	0.987	**0.989**	0.982
Spider Mites	0.994	0.994	**0.995**	0.985
Yellow Leaf Curl Virus	0.994	0.994	**0.995**	0.979
Healthy	0.994	0.987	**0.995**	0.985

CBAM attains the most stable gains on small and complex lesions.

Bold values indicate the best performance for each metric.

#### 3.5.3 Effectiveness of the above modules

Building on the above analysis, we conducted a systematic ablation that introduced transfer learning and then individual attention modules in a stepwise manner, and we compared mAP@0.5, parameter count, and GFLOPs (Giga Floating-point Operations Per Second) under a unified training schedule. Model 1 is the baseline network without transfer learning or attention modules. Model 2 applies transfer learning to the same baseline architecture. Model 3, 4, and 5 further incorporate different attention mechanisms on top of the transfer-initialized backbone, specifically GAM, SE, and CBAM, respectively. **Table 5** reports the full results. The baseline model, denoted Model 1, reached an mAP@0.5 of 0.959 with 11.14 million parameters and 28.7 GFLOPs. Adding transfer learning in Model 2 raised mAP@0.5 to 0.986 while keeping the model size and computation unchanged at 11.14 million parameters and 28.7 GFLOPs. On top of the transfer-initialized backbone, GAM increased the parameter count to 13.86 million and the computation to 34.2 GFLOPs, yet yielded only a modest accuracy of 0.976. SE preserved a lightweight budget at 11.14 million parameters and 28.7 GFLOPs and achieved 0.983, a balanced improvement over the baseline with minimal cost. CBAM delivered the best overall outcome, reaching 0.991 with 12.91 million parameters and 42.7 GFLOPs. Relative to the baseline, the final CBAM configuration improved mAP@0.5 by 3.2 percentage points, and relative to transfer learning alone, it added a further 0.5 percentage points. These results show that transfer learning provides the primary gain at constant compute, and that CBAM supplies the strongest attention-driven refinement with a moderate computational increase. The combined strategy strengthens detection accuracy, maintains a compact parameter budget compatible with edge deployment, and improves practical applicability for multi-target and multi-scale tomato leaf disease detection.

**Table 5:** Effects of transfer learning and attention modules on mAP@0.5, parameters and GFLOPs.

Model	Factors		mAP@0.5	Parameters/M	GFLOPs
	Transfer learning	Attention mechanism			
1	×	×	0.959	11.14	28.7
2	✓	×	0.986	11.14	28.7
3	✓	GAM	0.976	13.86	34.2
4	✓	SE	0.983	11.14	28.7
5	✓	CBAM	0.991	12.91	42.7

Transfer learning brings a clear boost and CBAM gives the best accuracy with acceptable cost.

## 4 Discussion

### 4.1 Placement of the CBAM Block

This study focused on leaf samples of four typical tomato diseases—bacterial spot, early blight, late blight, and leaf mold—and conducted systematic comparative experiments to determine the optimal insertion position of the CBAM attention module. Ultimately, CBAM was integrated after the C2f module in the backbone network, corresponding to the late-backbone pre-neck position. The core design logic addresses key challenges in tomato disease detection: the lesions exhibit high heterogeneity, ranging from fine-grained punctate spots (bacterial spot) to large-area mold-covered regions (late blight), and are susceptible to interference from leaf texture and illumination (Bhatia et al., 2020). CBAM’s channel and spatial attention mechanisms require precise alignment with feature optimization timing to adapt to these variations. Three candidate insertion points were evaluated: late-backbone (after the C2f module, before the neck), early-neck (after the first feature aggregation), and dual insertion (both positions). The primary goal was to balance detection accuracy for the target diseases with real-time deployment capability on edge devices. As the core of the YOLOv8 backbone, the C2f module aggregates multi-scale gradient flows via cross-layer connections, outputting features with shallow textures (suitable for fine-grained lesions like bacterial spot) and deep semantics (adapted to large-area lesions like late blight), providing a high-quality foundation for subsequent attention optimization (Zeiler and Fergus, 2014).

Integrating CBAM after the C2f module emerged as the optimal solution, with distinct advantages over the other two positions. This placement enables feature purification prior to multi-scale fusion: channel attention amplifies weights of disease-relevant channels while suppressing redundant healthy tissue signals, and spatial attention enhances lesion saliency (Lawal, 2021; Wang et al., 2021). It improves localization accuracy for both fine-grained and large-area lesions—experimental data show a 4.2% AP increase for bacterial spot and 5.7% higher localization accuracy for late blight—while only adding 8.3% to computational complexity and reducing inference speed by 3.1 FPS on edge devices (maintaining real-time performance). Early-neck insertion yielded comparable performance gains but introduced redundant attention on fused features, leading to diminishing marginal returns: boundary localization error for early blight’s concentric ring lesions was 11.3% higher, with 12.6% more additional computation. Dual insertion slightly improved small-target recall by 2.1% in dense lesion scenarios but caused a 23.5% surge in computational complexity, pushing inference speed below real-time thresholds and reducing detection accuracy by 3.4% in complex environments due to overfitting risks.

Overall, integrating CBAM after the C2f module is the optimal choice for detecting the four tomato diseases, offering comprehensive advantages in mechanism, performance, and efficiency. Mechanistically, it enables precise optimization after backbone feature extraction and before neck fusion, aligning with the C2f module’s ability to accommodate multi-scale lesions. In performance, it resolves blurred lesion localization and enhances detection accuracy for fine-grained, large-area, and complex-boundary lesions. Efficiently, it controls additional overhead, meeting edge device real-time requirements for practical scenarios like UAV field inspections. In contrast, early-neck insertion’s redundant gains and dual insertion’s poor cost-performance ratio make them unsuitable for target disease detection and real-world applications, so they were excluded from the final model.

### 4.2 Multi-target plant disease detection

Current deep learning–based multi-object detection methods have been widely applied in crop disease identification, weed detection, and pest monitoring. Nevertheless, spatial correlation among disease targets, the prevalence of small-scale or low-contrast lesions, and complex background interference remain persistent challenges for model design and optimization. Recent research has introduced Transformer-based architectures to model long-range spatial dependencies between co-occurring targets and has refined multi-scale feature fusion through FPN- and PANet-style necks to strengthen small-object sensitivity (Zayani et al., 2024). In parallel, self-supervised and semi-supervised learning alleviate label scarcity by mining structure from unlabeled images, while meta-learning improves adaptability across domains and seasons—together enhancing generalization and robustness under real-field variability. Despite these advances, many solutions incur substantial computational overhead or require large-scale curated data, which can limit real-time, on-device deployment in agricultural settings.

In this study, we address lesion overlap, scale variation, and background clutter in multi-target tomato leaf disease detection by proposing a lightweight YOLOv8s-CBAM model that integrates transfer learning with the Convolutional Block Attention Module (CBAM). YOLO v8s is adopted for its favorable accuracy–efficiency trade-off, while transfer learning injects strong priors from large-scale pretraining to accelerate convergence and stabilize performance under limited supervision. CBAM is inserted at the backbone–neck interface to provide complementary channel and spatial attention, enabling the detector to emphasize lesion-critical responses and improve localization stability for small or weak-texture targets without prohibitive cost. The pipeline is completed with a multi-target, multi-scale (MTMS) dataset assembled from public sources (PlantVillage), mosaic synthesis of dense scenes, and standard augmentations (rotation, flipping, cropping, brightness/contrast) to increase appearance diversity. We train with YOLO-formatted annotations and evaluate using precision, recall, F1-score, and mAP@0.5, including head-to-head comparisons with mainstream baselines (YOLO v5s, YOLO v8s, Faster R-CNN, SSD) under matched settings and ablation studies that isolate the contributions of transfer learning and CBAM. Empirically, the proposed approach improves feature representation and spatial localization of critical regions while maintaining a compact footprint (24.8 MB), thereby delivering accurate, generalizable, and real-time disease detection suitable for edge deployment in resource-constrained agricultural scenarios. Taken together, the gains originate from complementary mechanisms: transfer learning offers generic low-level priors that align with leaf texture and color cues, while CBAM supplies task-specific saliency that reinforces small-target localization under clutter; the MTMS dataset and mosaic synthesis expose the detector to dense, cross-scale compositions that mirror field conditions and make these architectural benefits observable in practice.

### 4.3 Application status of YOLO v8 in crop disease detection

With the advancement of precision agriculture and smart farming, deep learning-based object detection technologies have been widely applied in domains including crop disease diagnosis, pest monitoring, and yield estimation. YOLO v8, distinguished by its anchor-free detection head, C2f feature extraction module, and superior balance between accuracy and speed, has emerged as the preferred baseline model for real-time crop disease detection (Thakur et al., 2023). Currently, academic research regarding the application of YOLO v8 in agricultural scenarios primarily concentrates on the following three directions:

First, lightweight improvements oriented towards edge computing. Given the limited computational resources of agricultural detection devices including UAVs, inspection robots, and handheld terminals, extensive research is dedicated to reducing parameter count and computational complexity while maintaining detection accuracy (Bi et al., 2022). Wu et al. (2025) utilized MobileNet v3 to replace the backbone of YOLO v8 and constructed a lightweight D-YOLO model for strawberry health detection to effectively reduce parameters by 72.0%. Similarly, W. Liu et al. proposed a lightweight real-time recognition algorithm by incorporating a Lightweight Multi-Scale Module and applying pruning strategies to achieve a detection speed of 19.70 FPS on Jetson Nano devices (Liu et al., 2024). These improvements enable model deployment on embedded devices and realize offline real-time inference in field environments.

Second, feature enhancement targeting minute lesions and early-stage diseases. Crop diseases often manifest as tiny spots with indistinct textural features during the early stages and make them prone to information loss during downsampling. Researchers generally favor incorporating attention mechanisms and specialized convolution modules to address this issue. J. Wang et al (Wang et al., 2024) developed the YOLOv8-RCAA network for tea leaf disease detection by integrating the CBAM attention mechanism and RepVGG backbone to significantly enhance feature extraction capabilities for small targets. Furthermore, some studies attempt to introduce dynamic convolutions to enhance feature extraction capabilities for irregular or slender lesions. He et al. (2024) introduced Dynamic Snake Convolution into the C2f module to enhance the detection of irregular needle-shaped weed targets and effectively reduce false negative rates in complex field environments.

Third, robustness enhancement in complex field backgrounds. Drastic illumination changes, leaf occlusion, and background clutter including soil and weed interference in actual agricultural scenarios are primary factors constraining model performance. Current research hotspots focus on improving model generalization through multi-scale feature fusion and advanced architecture integration. Z. Liu et al. (2025) proposed the YOLO-BSMamba model which integrates a weighted Bidirectional Feature Pyramid Network BiFPN and the Mamba state space model to capture global contextual dependencies and thereby improve detection performance in complex backgrounds. Additionally, Y. Wang et al. (2024b) improved the Re-parameterized Generalized Feature Pyramid Network RepGFPN to enhance multi-scale feature fusion and effectively solve the problem of locating small leaf lesions under varying lighting and occlusion conditions (Cap et al., 2022).

In summary, although YOLO v8 possesses powerful foundational detection capabilities and current research has made significant progress in lightweighting and scenario-specific optimization, single-point improvements often struggle to balance global performance when facing the complex task of tomato disease detection characterized by multi-target multi-scale features and vast morphological differences. Compared to studies focusing solely on lightweight backbones like MobileNet v3 (Wu et al., 2025) or complex state-space models like Mamba (Liu et al., 2025), our proposed YOLOv8s-CBAM provides a more targeted solution for the specific “multi-target, multi-scale” (MTMS) nature of tomato lesions. While lightweight models (Liu et al., 2024) prioritize speed at the cost of spatial resolution for minute early-stage spots, our integration of the CBAM module at the backbone-neck interface ensures that both channel and spatial saliency are preserved without the heavy computational overhead of re-parameterized networks (He et al., 2024). Furthermore, unlike the RCAA network (Wang et al., 2024) which uses RepVGG, our approach leverages transfer learning from large-scale pretraining to mitigate the data scarcity common in specialized agricultural datasets. Many existing improvement schemes still leave room for improvement regarding stability when processing co-existing diseases and positioning extremely small targets. This is precisely the motivation behind the proposed improved YOLO v8s model which fuses transfer learning with the CBAM mechanism. It aims to systematically resolve the challenges of feature alignment and localization in multi-scale disease detection while maintaining lightweight advantages.

### 4.4 Limitation

The experimental results demonstrate that the proposed YOLOv8s-CBAM framework effectively balances detection precision and model compactness, particularly in challenging multi-target and multi-scale (MTMS) scenarios. This success aligns with recent advancements in attention-enhanced deep learning for robust agricultural classification (Hassan et al., 2025a; Eliwa and Abd El-Hafeez, 2025c), confirming that strategic feature recalibration is essential for managing the inherent variability of crop imagery. Furthermore, by providing an efficient solution for automated monitoring, this study contributes to the broader framework of deep learning for sustainable agriculture and food security (Eliwa and Abd El-Hafeez, 2025b).Despite the promising experimental results and structural innovations achieved in this study, several limitations and challenges remain for practical application and large-scale deployment. First, model training continues to rely on high-quality annotated data. Although the introduction of transfer learning alleviates some data scarcity issues, the difficulty in obtaining samples for rare diseases or early-stage lesions may still impact the model’s generalization capability. Second, the robustness of the model in extremely complex environments requires further improvement. In scenarios involving strong illumination, shadows, leaf overlap, or blurred lesion boundaries, issues such as reduced detection confidence or bounding box drift may still occur (Tang et al., 2020). Third, although the model has been optimized for lightweight deployment, the inference speed remains slightly lower than that of some extremely compressed models when processing ultra-high-resolution or densely populated multi-target images, limiting its suitability for real-time detection tasks with stringent latency requirements. In addition, the current model has been validated only on tomato leaf diseases and has not yet been generalized to other crops or diseases affecting stems, roots, or fruits (Espejo-Garcia et al., 2020). Future work should extend the application scope through cross-crop transfer learning and broader validation.

To enhance model practicality and intelligence, future research will advance under real-time edge constraints via a unified framework of data efficiency, model optimization, task coupling, sensing enhancement, and end-to-end systemization. Use few-shot + incremental/continual learning, augment scarce class data with GANs and diffusion models to mitigate class imbalance and improve emerging disease recognition. For efficiency, compress networks through pruning, knowledge distillation, quantization, etc., paired with tools like ONNX for lightweight real-time edge deployment. Build a multi-task framework with a shared backbone + multi-head decoder to unify lesion detection, classification, and severity assessment, boosting decision robustness. Fuse multimodal data at the perception level, leverage semi/self-supervised learning to reduce annotation costs and enhance domain generalization. Ultimately, integrate UAVs with edge-cloud architecture for data collection, real-time inference and early warning, and GIS visualization, constructing a detection and early warning platform to support precision intelligent agriculture. Specifically, we will explore the frontiers of image super-resolution (Hassan et al., 2025b) to further improve the visibility of microscopic early-stage lesions and investigate semantic segmentation techniques (Eliwa and Abd El-Hafeez, 2024) to provide pixel-level quantification of microbial alterations, ensuring the sustained evolution of the proposed system.

## 5 Conclusion

Evidence converges across metrics, visual diagnostics, and ablation experiments. The proposed design enhances lesion-critical representations and enables faster, more stable optimization under limited supervision. In response to the challenges of multi-target, multi-scale, and complex background detection in tomato leaf disease identification, this study proposes a lightweight object detector, YOLOv8s-CBAM, that integrates the CBAM attention mechanism with transfer learning. During dataset construction, publicly available data from PlantVillage and AI Challenger were curated and combined with image mosaicking and multi-scale augmentation to form a corpus exhibiting dense targets, broad scale variation, and cluttered field-like backgrounds. Labels follow the YOLO format with a stratified train/validation/test split, and class balance is improved through controlled sampling and brightness, contrast, rotation, flip, and crop transformations to increase appearance diversity and stabilize training for minority disease classes. In model design, YOLO v8s is adopted for its accuracy–efficiency trade-off, CBAM is inserted at the backbone–neck interface to deliver complementary channel- and spatial-level focus on lesion regions, and transfer learning initializes the backbone with large-scale pretrained weights to accelerate convergence and strengthen generalization under limited supervision. Training and evaluation adhere to standard practice with precision, recall, F1-score, and mAP@0.5; head-to-head comparisons are conducted against YOLO v5s, the YOLO v8s baseline, Faster R-CNN, and SSD under matched settings, and ablation studies isolate the individual effects of transfer learning and attention, including alternative attention variants for completeness. Experimental results show that YOLOv8s-CBAM delivers mAP@0.5 = 99.1%, F1 = 97.0%, precision = 96.9%, and recall = 97.3%, while retaining a compact 24.8 MB footprint that supports real-time, on-device deployment. The ablations confirm the effectiveness of both the attention mechanism and the transfer strategy; per-class analysis indicates mAP > 0.995 on complex categories including spider mite damage and leaf mold, reflecting strong localization stability and generalization. Remaining limitations involve the dependence on high-quality annotations, robustness under extreme illumination and occlusion, and cross-crop adaptability. Future work will emphasize few-shot adaptation to emerging diseases, compression and hardware-aware inference for faster edge execution, multimodal integration of visible, infrared, and hyperspectral cues, and coordinated edge–device deployment, with the long-term goal of translating accurate detection into a scalable early-warning platform for precision and intelligent agriculture.

## Data Availability

The original PlantVillage dataset used in this study is publicly available. The processed dataset and annotation files generated during the current study are available from the corresponding author upon reasonable request.
